# Investigation of *ITGB3* Heterogeneity to Overcome Trastuzumab Resistance in HER2-Positive Breast Cancer

**DOI:** 10.3390/biology14010009

**Published:** 2024-12-25

**Authors:** Asiye Busra Boz Er

**Affiliations:** Department of Medical Biology, Faculty of Medicine, Recep Tayyip Erdogan University, 53100 Rize, Turkey; asiyebusra.bozer@erdogan.edu.tr

**Keywords:** HER2-positive breast cancer, *ITGB3*, TGF-β signalling, cilengitide, tumour heterogeneity, trastuzumab resistance

## Abstract

HER2-positive breast cancer has an aggressive tumour progression among breast cancers characterized by the overexpression of HER2. Trastuzumab is an FDA-approved drug and has significantly improved outcomes for patients; however, drug resistance remains a major challenge. Tumour heterogeneity, describing genetic, epigenetic, and phenotypic differences within and between tumours, complicates tumour treatment and contributes to drug resistance. Understanding the mechanisms underlying Trastuzumab resistance, such as tumour heterogeneity, is crucial for developing new and effective therapeutic strategies. This study investigates the role of *ITGB3* heterogeneity in Trastuzumab resistance, focusing on its impact on TGF-β signalling and migration marker response. It also evaluates the potential of combining Trastuzumab with the integrin β3 inhibitor cilengitide to overcome resistance associated with *ITGB3* levels. Trastuzumab-resistant HER2-positive HCC1954 and SKBR3 breast cancer cell lines were generated and analysed for *ITGB3* expression heterogeneity. The impact of *ITGB3* on TGF-β-responsive genes *(WWP1*, *CARM1*, *RASGRP1*, *THBS1*, *KCTD5*, *SGCA*, *EIF3S6*, *MCAM*, *FXR2*, *MTMR3*, *SOCS3*, *SLC2A4RG*, *MMP2*, *MMP9*, and *HSP47)* and cell migration (*Col4a1*, *fibronectin*, *ICAM1*, *Timp2*, and *vimentin*) was analysed using luciferase reporter assays and real-time PCR. The effects of combined treatment with Trastuzumab and cilengitide were also evaluated via wound closure assay. *ITGB3* expression varied significantly among resistant clones, correlating with increased expression of TGF-β-responsive genes and enhanced migration markers. Combined treatment with Trastuzumab and cilengitide significantly reduced TGF-β signalling and migration-related gene expression, particularly in high *ITGB3*-expressing cells. *ITGB3* plays a critical role in Trastuzumab resistance through the modulation of TGF-β signalling, migration, and contributing to tumour heterogeneity. Targeting ITGβ3, alone or in combination with cilengitide, offers a promising strategy to resensitize resistant HER2-positive breast cancer cells to Trastuzumab. These findings provide valuable insights into the mechanisms of Trastuzumab resistance and suggest potential therapeutic avenues for improving patient outcomes.

## Simple Summary

HER2-positive breast cancer is an aggressive form of cancer and is often resistant to trastuzumab, a key FDA-approved therapy. The following study explores how the heterogeneity of *ITGB3* contributes to trastuzumab resistance by affecting TGF-β signaling and migration markers. Using resistant breast cancer cell lines, researchers found that *ITGB3* expression varied widely and was correlated with increased migration and signaling activity. Combining trastuzumab with cilengitide, an integrin β3 inhibitor, reduced these effects, particularly in cells with high *ITGB3* levels. Targeting ITGβ3 could aid in overcoming trastuzumab resistance and improve treatment outcomes for HER2-positive breast cancer patients.

## 1. Introduction

HER2-positive breast cancer is a subtype characterized by the overexpression of the human epidermal growth factor receptor 2 (HER2), which drives aggressive tumour growth and poor clinical outcomes [1]. The development of targeted therapies, such as Trastuzumab, a monoclonal antibody that binds to the extracellular part of HER2, has significantly improved the prognosis for patients with HER2-positive breast cancer [2]. Even though Trastuzumab shrinks tumour well at first, many patients eventually develop resistance, causing the disease to relapse and metastasise. In this aspect, understanding the mechanisms underlying Trastuzumab resistance is crucial for developing new therapeutic strategies to improve patient outcomes.

Current treatment options for Trastuzumab-resistant breast cancer face several significant limitations [1]. Resistance often arises through complex mechanisms, such as the activation of alternative signalling pathways (e.g., PI3K/AKT, Hedgehog, Notch) and changes in receptor expression, which limit the effectiveness of therapies targeting HER2 alone. While subsequent HER2-targeted therapies like pertuzumab, T-DM1, and lapatinib offer additional options, their efficacy is frequently short-lived, particularly in advanced cases, as resistance can develop quickly [3]. Additionally, intensified treatment regimens combining targeted therapies with chemotherapy increase the risk of adverse side effects, impacting patient quality of life and treatment adherence. Tumour heterogeneity further complicates outcomes, as it enables subpopulations of cancer cells to evade therapy and survive within the tumour microenvironment [4]. Moreover, resistance is often associated with cancer stem cell characteristics and epithelial-to-mesenchymal transition (EMT), which are not adequately addressed by current therapies, leading to persistent challenges in managing recurrence and metastasis. These limitations highlight the need for future approaches that combine HER2-targeted therapies with treatments aimed at overcoming resistance pathways to improve efficacy and durability in Trastuzumab-resistant breast cancer.

Integrins are transmembrane receptors that facilitate cell–extracellular matrix (ECM) adhesion, playing vital roles in various cellular processes, including migration, invasion, and survival [5]. Integrin β3, a subunit of the RGD binding integrins, has been implicated in cancer progression and resistance to therapy. ITGβ3 can influence several downstream signalling pathways, including the TGF-β pathway, which is known for its dual role in cancer [6]. In the early stages of tumorigenesis, TGF-β acts as a tumour suppressor; however, in advanced stages, it promotes tumour progression, metastasis, and therapy resistance [7]. The interaction between ITGβ3 and the TGF-β signalling pathway is a key area of interest in understanding the mechanisms of Trastuzumab resistance.

The TGF-β signalling pathway plays a vital role in controlling cell proliferation, differentiation, and migration. In the context of cancer, TGF-β signalling can trigger epithelial–mesenchymal transition (EMT), a process wherein epithelial cells transition to a mesenchymal phenotype, thereby gaining increased migratory and invasive capacities [8,9]. The upregulation of TGF-β-responsive genes, including WWP1, CARM1, RASGRP1, and THBS1, has been linked to heightened malignancy and resistance to therapeutic interventions. This study delves into the interplay between ITGB3 expression and TGF-β signalling in Trastuzumab-resistant HER2-positive breast cancer cells. Emerging research emphasizes the potential of targeting integrins to overcome drug resistance in cancer. Cilengitide, an inhibitor of integrin β3, has demonstrated effectiveness in disrupting integrin-mediated signalling pathways, thereby limiting cancer cell motility and invasiveness [10]. Despite its hopeful results in preclinical studies, cilengitide has been shown to be less effective than expected in clinical studies. However, using integrin inhibitors together with existing treatments like Trastuzumab has potential to increase the efficiency of the current therapies and could be a strong and effective way to overcome treatment resistance.

Cancer’s complexity is driven by genetic changes that enhance proliferation, evade cell death, and promote metastasis [11]. It evolves dynamically, leading to significant tumour heterogeneity, posing challenges for effective treatment and contributing to drug resistance [12]. Tumour heterogeneity includes spatial differences within and between tumours and temporal changes over time [13]. This heterogeneity affects protein and gene expression levels, leading to varied cellular behaviours and drug responses. As a result, different subclones may exhibit distinct drug sensitivities, complicating treatment strategies. Tumour heterogeneity allows subclones with inherent drug resistance to survive treatment, and initially sensitive cells can develop resistance over time through mutations and pathway activation. Accurate evaluation of tumour heterogeneity is crucial for effective treatment. Personalized therapies targeting multiple pathways and subclonal populations may overcome resistance [14]. Advanced diagnostic techniques and combination therapies hold promise for better outcomes. Addressing this complexity through personalized and combination therapies can improve treatment efficacy and patient prognosis.

In this study, Trastuzumab-resistant cell lines were generated from HCC1954 and SKBR3, two HER2-positive breast cancer cell lines, and the heterogeneity in ITGB3 expression among these resistant clones was examined. The impact of ITGB3 expression on the activation of TGF-β-responsive genes and cell migration was analysed. Furthermore, the therapeutic potential of combining Trastuzumab with cilengitide in modulating these pathways and overcoming resistance was evaluated.

Cell migration and epithelial-to-mesenchymal transition (EMT) are crucial processes in the progression and metastasis of breast cancer. EMT enables epithelial cells to acquire mesenchymal traits, which enhances their motility, invasiveness, and resistance to apoptosis, key characteristics that facilitate cancer metastasis. During EMT, cells lose their epithelial markers, such as E-cadherin, and gain mesenchymal markers, like vimentin and fibronectin, which contribute to enhanced cell migration and the ability to invade surrounding tissues [15]. This transition not only promotes local invasion but also primes cancer cells for entry into the bloodstream or lymphatic system, enabling distant colonization of secondary organs. In HER2-positive breast cancer, the activation of EMT and migration pathways is often driven by growth factor signalling, including TGF-β, which has been shown to upregulate key EMT markers such as Snail, Twist, and Zeb1 [16]. The role of EMT in breast cancer metastasis underscores its significance as a therapeutic target. By disrupting EMT and migration processes, such as through the inhibition of integrin-mediated signalling or TGF-β pathways, it may be possible to prevent metastasis and improve patient outcomes in HER2-positive breast cancer [17]. Understanding and targeting these pathways are crucial for developing more effective treatments to counteract the aggressive and metastatic nature of this cancer subtype.

Our findings provide insights into the molecular mechanisms underlying Trastuzumab resistance, highlighting the role of ITGB3 in regulating TGF-β signalling and migration-related pathways. The results suggest that targeting ITGB3, alone or in combination with cilengitide, may offer a promising strategy to resensitize resistant HER2-positive breast cancer cells to Trastuzumab. This study paves the way for further research and potential clinical applications to improve outcomes for patients with Trastuzumab-resistant HER2-positive breast cancer.

## 2. Methods

### 2.1. Cell Culture, Drug Treatment

**Figure 1:**
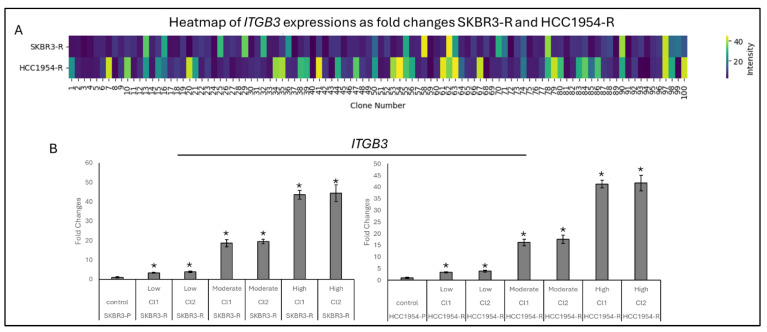
*ITGB3* shows heterogeneity in resistant cell populations (**A**). Heatmap shows heterogeneity of *ITGB3* expression as fold changes for 100 colonies from single cells for SKBR3-R and HCC1954-R cells. (**B**) Bar graph of chosen colonies *ITGB3* expression levels for SKBR3 and HCC1954 from low, moderate, high and parental groups. Two-way ANOVA with a Tukey’s post hoc test was used. Parental (P) and resistant (R) cell lines are indicated. * *p* ≤ 0.05, *n* = 3 ± SD.

HCC1954 (ATCC Cat# CRL2338) and SKBR3 (ATCC Cat# HTB30) are HER2-positive and EGFR-high/ERBB2-amplified breast cancer cell lines known for their capability to develop Trastuzumab resistance and were purchased from ATCC (Cheyenne, WY, USA) [18,19]. The cell lines were grown in DMEM (Gibco, Carlsbad, CA, USA) medium supplemented with 10% FBS, 1% sodium pyruvate, and 2 mM L-glutamine. HCC1954 and SKBR3 Trastuzumab-resistant cell lines were generated by exposing the cells to increasing doses (0.1–10 μM) of Trastuzumab (Cat no: A2007, Selleckchem, Houston, TX, USA) for 3 months [20,21,22,23]. To confirm the acquisition of resistance in the newly generated cells, MTT viability assays were performed, and the IC_50_ results of the cells were analysed. Chronic exposure to Trastuzumab resulted in the development of resistance, with the cells exhibiting higher IC_50_ levels. For SKBR3 cells, the IC_50_ increased from approximately 0.2 to 2.6 μM, and for HCC1954 cells, it increased from approximately 0.3 to 2.4 μM. The HCC1954 and SKBR3 cells were seeded into six-well plates (2.2 × 10^5^/well) at a confluency of 40–60% and transfected 24 h later with a total of 2 μg of plasmid DNA per well using lipofectamine 2000 (Waltham, MA, USA), according to the manufacturer’s instructions. The IC_50_ levels for cilengitide monotherapy, as reported in our previous paper, were 0.8 μM for SKBR3-P and 0.6 μM for SKBR3-R. Similarly, for HCC1954 cells, the IC_50_ values were 0.6 μM for HCC1954-P and 0.7 μM for HCC1954-R [20]. Following IC_50_ calculations, single cells from the resistant pool were isolated and seeded into 12-well plates to form uniform colonies and *ITGB3* gene expression levels characterized, as shown in Figure 1.

### 2.2. Luciferase Reporter Assay

Cells were transfected with TGF-β-responsive reporter plasmids, including 3TP-LUX (driven by the PAI-1 promoter) and SBE-LUC (containing a Smad binding element), to evaluate the activation of the TGF-β pathway. To maintain a consistent plasmid concentration across all wells, the total amount of plasmid was equalized using an empty-FLAG vector. Additionally, cells were transfected with the pCMV-β-Gal plasmid to standardize transfection efficiency across the experiments. The SBE-LUC and pCMV-β-Gal plasmids were generously provided by Dr. Talat Nasim from the University of Bradford, UK, while the p3TP-LUX plasmid was kindly donated by Dr. Joan Massague and Dr. Jeff Wrana (Addgene plasmid # 11767) [24,25].

Twenty-four hours post-transfection, cells were lysed using a reporter lysis buffer (Promega, Cat. No. E4030). Luciferase activity was then measured using a luminometer (Fluoroskan Ascent FL-Thermo Scientific, Waltham, WA, USA) immediately following the addition of the luciferase assay system substrate, luciferin. To account for variations in transfection efficiency, luciferase activity was normalized against β-galactosidase activity.

The β-galactosidase activity was quantified by measuring absorption at 405 nm after incubation with a solution containing Ortho-Nitrophenyl-β-galactoside (ONPG) (4 mg/mL), β-mercaptoethanol, and Z buffer (composed of Na2HPO4.7H2O (0.06 M), NaH2PO4.H2O (0.04 M), 1M KCl (0.01 M), and 1M MgSO4 (0.001 M)). The reaction was stopped by adding a 1 M Na_2_CO_3_ buffer. The resulting luciferase assay data were then normalized to the β-galactosidase assay results to ensure consistent transfection efficiency across all samples.

### 2.3. Real-Time PCR

Cells were lysed, and RNA was extracted using a Qiagen RNAeasy kit (Qiagen, Venlo, The Netherlands) according to the manufacturer’s guidelines. For cDNA synthesis, the Biorad iScript Reverse Transcription Supermix (Biorad, Hercules, CA, USA) for RT-qPCR was utilized. Quantitative real-time PCR (RT-qPCR) was performed using the iTaq Universal SYBR Green One-Step Kit (Biorad, Hercules, CA, USA), with Ct values measured on an Applied Bioscience ABI 7500 Real-Time Instrument (Waltham, MA, USA) equipped with 7500 Software v1. The amplification protocol consisted of an initial denaturation step at 95 °C for 10 s, followed by 40 cycles of 60 °C for 1 min. The melting curve analysis included a denaturation phase at 95 °C for 15 s, annealing at 60 °C for 1 min, and final elongation at 95 °C for 15 s.

Primers used for RT-qPCR were custom-designed using the Primer3 software and obtained from Macrogen (Supplementary Table S1). Each primer set and sample were tested in triplicate to ensure accuracy, and the entire experiment was repeated three times with different biological samples to confirm reproducibility.

To analyse the RT-qPCR data, Ct values of the target genes were normalized against the housekeeping gene GAPDH. The normalized Ct values (ΔCt) were calculated as ΔCt = Ct (target gene average) − Ct (housekeeping gene average). Relative gene expression was determined using the 2^−∆Ct^ method, and fold changes were calculated using the 2^−ΔΔCt^ method: 2^−∆Ct^ (sample)/2^−∆Ct^ (control).

### 2.4. Heat Map Generation

Heatmaps were constructed using Python with the support of libraries such as NumPy, Matplotlib, and Seaborn. The Python code was executed in the Google Colab environment to facilitate the visualization. Initially, the data—comprising gene names (as row labels), cell line identifiers (as column labels), and their respective quantitative values—were formatted into a NumPy array.

To visualize these data, Seaborn’s heatmap function was employed, which allowed for clear representation of the data matrix. A diverging colour palette was selected to distinctly highlight positive and negative values. The axes were appropriately labelled to ensure clarity, and Matplotlib was utilized to display the final heatmap.

### 2.5. Volcano Plot Generation

To create a volcano plot, a dataset is first collected that includes fold changes and *p*-values for two comparisons, such as SBE-LUC and 3TP-LUC. The data are formatted into a table with columns for the cell line name, condition labels (e.g., CL1, CL2), expression levels (e.g., low, moderate, high), fold change values, and *p*-values for both comparisons. Next, log transformations are performed on the data to compute the log2 fold change and −log10 of the *p*-values for each comparison.

Using libraries like matplotlib and seaborn, the plot is set up by defining regions with background shading to visually distinguish different expression levels: low, moderate, and high. Scatter plots are created for the data points of both comparisons, with different colours used to differentiate between them. Annotations are then added to each point to indicate the condition and expression level, with positions adjusted to avoid overlap.

### 2.6. Wound Healing Assay

Cells were seeded at 90% confluency and incubated overnight at 37 °C in a CO_2_ incubator. Once the cells reached 100% confluency, wounds were created using a pipette tip, and the cells were washed three times with PBS to remove debris. Treatments were applied at IC50 concentrations for cilengitide monotherapy, Trastuzumab monotherapy, and their combination. Images of the wounds were captured at 0 h and after 72 h of incubation. Wound healing rates were analysed using the ImageJ software (1.53e, Java-1.8.0_172).

### 2.7. Statistical Analysis

Statistical analysis was conducted using the GraphPad software (10.2.1). A two-way ANOVA was utilized to evaluate variance, followed by a Tukey post hoc test to assess pairwise comparisons for statistical significance. Results were deemed significant at *p* ≤ 0.05. Error bars denote the mean ± standard deviation (SD) derived from three independent experiments, each performed in triplicate.

## 3. Results

### 3.1. ITGB3 Expression Has Variety Between Different Clones

**Figure 2:**
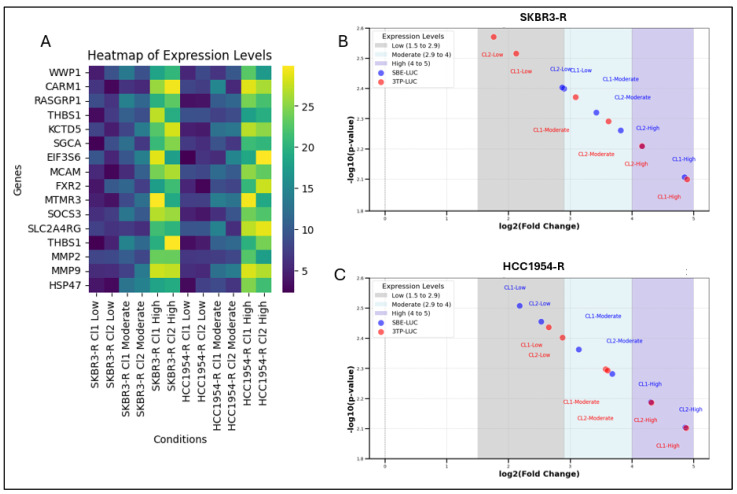
The TGF-β-responsive genes exhibited elevated expression levels in SKBR3-R and HCC1954-R clones with high ITGB3 expression. (**A**) Heatmap of TGF-B-responsive gene expression levels as fold changes in SKBR3-R and HCC1954-R in the presence of low, moderate and high ITGB3 expression. SBE-LUC and 3TP-LUC TGF-B reporter response to presence of low, moderate and high ITGB3 expression in (**B**) SKBR3-R and (**C**) HCC1954-R. Resistant (R) cell lines are indicated. Two-way ANOVA with a Tukey’s post hoc test was used *p* ≤ 0.05, *n* = 3.

Trastuzumab-resistant HCC1954 and SKBR3 cell line pools were developed by exposing the cells to increasing concentrations of Trastuzumab (0.1–10 μM) over a period of three months. Following this exposure, the cells were collected and lysed with lysis buffer, and ITGβ3 expression was analysed via Western blot. ITGβ3 expression was increased in the resistant cell pool of HCC1954 and SKBR3-R cells (Figure S1). After confirmation of ITGβ3 expression, single cells from the resistant pools were isolated and seeded into 12-well plates to form uniform colonies. From the resulting drug-resistant populations, 100 colonies were generated from single cells. RNA was isolated from these colonies, and real-time PCR was performed to detect *ITGB3* levels. Interestingly each cell taken from HCC1954 and SKBR3-R pools has significantly increased *ITGB3* levels (Figure S2). The analysis of *ITGB3* gene expression as fold changes revealed a significant increase in 25 colonies compared to the parental cells, with expression levels below 5-fold. In 45 colonies, ITGB3 expression was elevated between 5- and 10-fold, and in 30 colonies, it exceeded 10-fold, with the highest recorded increase being 45-fold (Figure 1). Based on these expression levels, the colonies were categorized into low, moderate, and high groups. Two colonies were selected from each group to analyse the heterogeneity of *ITGB3* expression on migration, TGF-β pathway activation, and response to drug treatment (Figure 2).

### 3.2. TGF-B Signalling Associated with ITGB3 Gene Expression Levels

TGF-β levels are known to be elevated in HER2-positive Trastuzumab-resistant cell lines. Several TGF-β-responsive genes, including WWP1, CARM1, RASGRP1, THBS1, KCTD5, SGCA, EIF3S6, MCAM, FXR2, MTMR3, SOCS3, SLC2A4RG, MMP2, MMP9, and HSP47, have been previously identified in other cancer cell types such as A549 (lung adenocarcinoma) and HPL1D (lung epithelial cells) [26]. However, the expression patterns and responses of these genes have not been explored in HER2-positive Trastuzumab-resistant breast cancer cells. This study focuses on examining the relationship between the expression of these specific genes and *ITGB3* heterogeneity in Trastuzumab-resistant HER2-positive breast cancer cells [8].

The TGF-β-responsive genes *WWP1*, *CARM1*, *RASGRP1*, *THBS1*, *KCTD5*, *SGCA*, *EIF3S6*, *MCAM*, *FXR2*, *MTMR3*, *SOCS3*, *SLC2A4RG*, *MMP2*, *MMP9*, and *HSP47* were found to be significantly upregulated in Trastuzumab-resistant SKBR3 and HCC1954 cell lines across all *ITGB3* expression levels—low, moderate, and high (Figure S3). Notably, in cells with low and moderate *ITGB3* expression, the upregulation of these genes did not exceed a fold change of 15. However, in cells characterized by high *ITGB3* expression, the fold change in gene expression was consistently greater than 15 (Figure 2). These findings suggest a clear association between elevated *ITGB3* levels and an amplified TGF-β response.

To confirm these findings, luciferase reporter assay was conducted to analyse TGF-β activity. Trastuzumab-resistant SKBR3 and HCC1954 cell lines with low, moderate, and high ITGB3 expression levels transfected with TGF-β reporter plasmids and β-Gal expressing plasmids. Further, 24 h later, luciferase and β-Gal activity was measured. Results were normalized with β-Gal expression. The volcano plots provide a comparative analysis of gene expression fold changes (log2) and statistical significance (−log10 *p*-value) in response to different ITGB3 expression levels. In SKBR3-R cells, TGF-β-responsive reporters SBE-LUC and 3TP-LUC showed significant upregulation in clones with high ITGB3 expression (log2 fold change > 4, *p* < 0.01), while moderate ITGB3 expression also caused notable changes (Figure 2B). Low ITGB3 expression led to modest changes in gene expression. Similarly, in HCC1954-R cells, high ITGB3 expression resulted in significant upregulation of multiple genes, while moderate expression levels induced appreciable changes, and low ITGB3 expression caused fewer significant alterations (Figure 2C).

These results indicate that ITGB3 expression modulates TGF-β response in both cell lines, with variations depending on the cell line, suggesting its impact on TGF-β signalling pathways and highlighting it as a potential therapeutic target.

**Figure 3:**
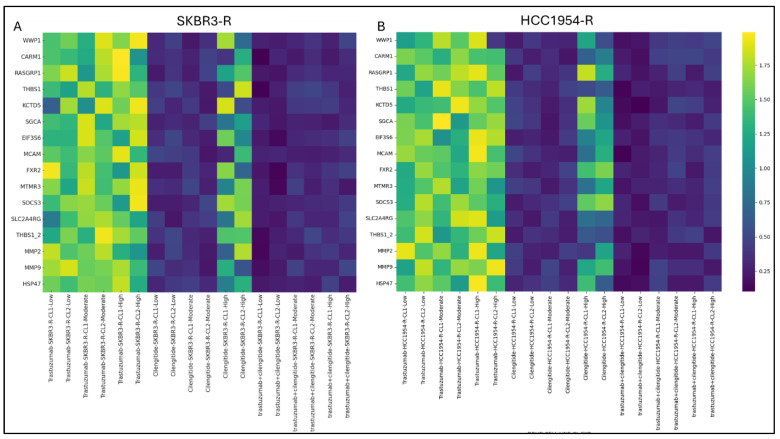
Cilengitide monotherapy decrease TGF-B responsive expressions only low and moderate ITGB3 expressing cells while Trastuzumab+cilengitide decreased low, moderate and high ITGB3 expressing groups in SKBR3-R and HCC1954-R cells. (**A**) Heatmap of TGF-B responsive gene expression levels as fold changes in the presence of low, moderate and high ITGB3 expression in the treatment of cilengitide and trastuzumab monotherapy and combination in (**A**) SKBR3-R and (**B**) HCC1954-R. Two-way ANOVA with a Tukey’s post hoc test was used. Parental (P) and resistant (R) cell lines are indicated. *p* ≤ 0.05, *n* = 3.

Given the observed association between *ITGB3* expression and TGF-β activity (Figure 2B,C), we investigated the therapeutic potential of the ITGβ3 inhibitor, cilengitide, and its effect on TGF-β signalling in Trastuzumab-resistant HER2-positive breast cancer cell lines, SKBR3-R and HCC1954-R. TGF-β levels were found to be highest in cells treated with Trastuzumab monotherapy (at the IC_50_ concentration) for 24 h, regardless of low, moderate, or high ITGB3 expression levels (Figure 3A,B).

In the cilengitide monotherapy group, a significant reduction in TGF-β-responsive genes was observed in cells with low to moderate ITGB3 expression. However, cilengitide alone did not reduce TGF-β-responsive genes in cells with high ITGB3 expression (Figure 3A,B). In contrast, the combination of Trastuzumab and cilengitide significantly suppressed TGF-β signalling across all ITGB3 expression levels—low, moderate, and high—in both SKBR3-R and HCC1954-R cell lines. Only statistically significant results are presented in the heatmap (Figure S4)

### 3.3. ITGB3 Expression Linked with Migration

**Figure 4:**
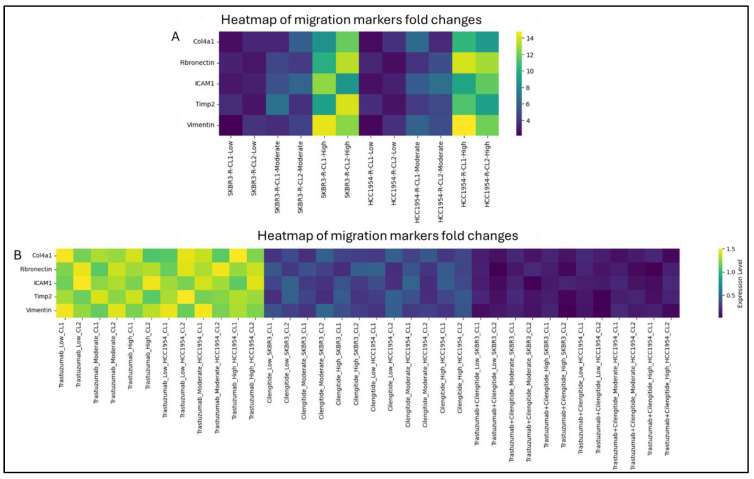
Migration markers expressions correlated with ITGB3 levels and trastuzumab+cilengitide combination significantly decrease migration markers. (**A**) Migration marker expressions in low, moderate and high *ITGB3* expressing cells of SKBR3-R and HCC1954-R. (**B**) Migration marker expressions in low, moderate and high *ITGB3* expressing cells of SKBR3-R and HCC1954-R in the presence of cilengitide and trastuzumab monotherapy and combination. Two-way ANOVA with a Tukey’s post hoc test was used. Parental (P) and resistant (R) cell lines are indicated. *p* ≤ 0.05, *n* = 3.

The TGF-β pathway is critically involved in regulating cell migration. To assess how varying levels of ITGB3 expression impact migratory behaviour, we analysed the expression of key migration markers—Col4a1, fibronectin, ICAM1, Timp2, and vimentin. Among the cells with low ITGB3 expression, migration marker levels showed the smallest fold change. In contrast, cells with moderate ITGB3 expression exhibited a noticeable increase, while those with high ITGB3 expression demonstrated the greatest upregulation of migration markers in both SKBR3-R and HCC1954-R cell lines (Figure 4A). These results highlight a strong correlation between higher ITGB3 expression levels and increased migratory capacity (see related *p*-values in Figure S5).

To further explore the effects of inhibiting ITGB3 expression on migration markers, SKBR3-R and HCC1954-R cells were treated with IC50 concentrations of cilengitide or Trastuzumab as monotherapies, as well as a combination treatment at a 1:1 IC50 ratio, for 24 h across groups with varying ITGB3 expression levels (Figure 4B). In the Trastuzumab monotherapy group, migration marker expression was consistently higher compared to the cilengitide monotherapy group across all ITGB3 expression levels. However, the combination of Trastuzumab and cilengitide led to the lowest migration marker expression levels (see related *p*-values in Figure S5).

These findings suggest that cilengitide as a single agent may offer greater therapeutic benefits than Trastuzumab alone in treating resistant cell lines, regardless of ITGB3 expression levels. Moreover, the combination of Trastuzumab and cilengitide appears to have a synergistic or additive effect in reducing migration, pointing to its potential in preventing metastasis.

**Figure 5:**
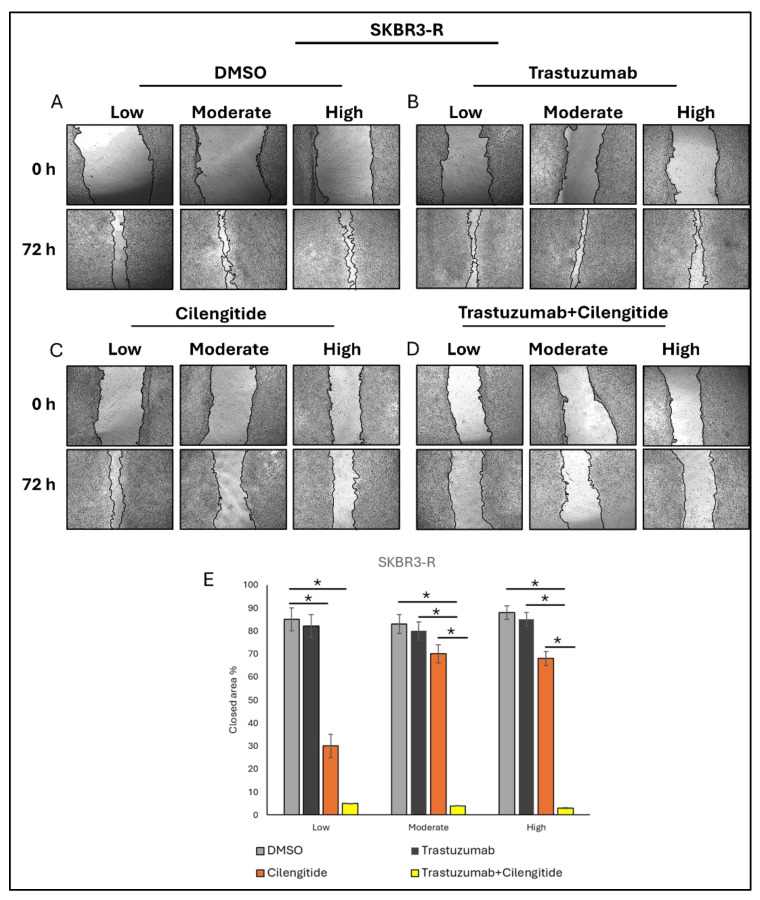
The combination of trastuzumab and cilengitide decreases wound closure in low, moderate, and high ITGB3-expressing SKBR3-R cells. Representative images of wound closure are shown for: (**A**) DMSO control, (**B**) trastuzumab monotherapy, (**C**) cilengitide monotherapy, and (**D**) trastuzumab+cilengitide combination treatment at 0 and 72 h. (**E**) Graph depicting the percentage of wound closure for each treatment group. Two-way ANOVA with a Tukey’s post hoc test was used * *p* ≤ 0.05, *n* = 3, SD±.

A scratch wound healing assay was conducted to assess how gene expression variations influence the migration ability of SKBR3-R cells and their response to trastuzumab, cilengitide, and their combination. After creating the scratch, cells were photographed and incubated for 72 h with inhibitors or control treatments. Wound closure areas were measured and analysed using ImageJ software. The results showed that in low ITGB3 expression cells, the wound closure percentage was approximately 85% in the control group, with no significant difference observed in Trastuzumab monotherapy compared to the DMSO control. Cilengitide monotherapy significantly reduced wound closure to approximately 30%, and the combination of Trastuzumab and cilengitide further reduced closure to just 5%. The combination treatment significantly inhibited migration compared to all other conditions, while cilengitide monotherapy also reduced migration compared to both DMSO and trastuzumab monotherapy (Figure 5).

In moderate *ITGB3* expression cells, the wound closure percentage was approximately 85% in the control group, similar to low *ITGB3* expression cells. Trastuzumab monotherapy reduced wound closure slightly to approximately 80%, while cilengitide monotherapy reduced it further to 70%. The combination therapy drastically reduced wound closure to around 4%. However, no significant differences were observed between trastuzumab monotherapy, cilengitide monotherapy, and the DMSO control. Similarly, in high *ITGB3* expression cells, the wound closure percentage was approximately 85% in the control group, consistent with both low and moderate *ITGB3* expression cells. Trastuzumab monotherapy reduced wound closure to approximately 80%, while cilengitide monotherapy reduced it to around 70%, and the combination therapy further reduced it to just 3%. No significant differences were observed between Trastuzumab monotherapy, cilengitide monotherapy, and the DMSO control in this group as well (Figure 5).

## 4. Discussion

HER2-positive breast cancer, characterized by the overexpression of the HER2 receptor, poses significant therapeutic challenges due to the frequent development of resistance to Trastuzumab. While Trastuzumab has markedly improved clinical outcomes for many patients, its effectiveness is often diminished over time due to resistance mechanisms, leading to relapse and disease progression. Understanding these resistance mechanisms is crucial for developing new strategies to enhance treatment efficacy and improve patient outcomes.

Integrin β3 regulates TGF-β signalling and cell migration through several mechanisms [17]. As a key component of integrins, ITGB3 interacts with the extracellular matrix (ECM), facilitating bidirectional signalling that influences cell behaviour in response to TGF-β stimulation. Integrin β3 can cooperate with TGF-β receptors to activate the SMAD pathway, promoting phosphorylation of SMAD2/3 and the transcription of TGF-β-responsive genes involved in migration, epithelial–mesenchymal transition (EMT), and fibrosis [27]. Additionally, integrin β3 influences non-canonical TGF-β signalling pathways by activating small GTPases like RhoA, Rac1, and Cdc42, which are critical for cytoskeletal reorganization and migration [28]. ITGB3 also plays a role in ECM remodelling by facilitating the expression of matrix metalloproteinases (MMPs) that degrade the ECM, aiding in cell movement. Furthermore, integrin β3′s interaction with TGF-β receptors can enhance their activation and trafficking, sustaining TGF-β signalling, especially in migration and invasion processes. Through its regulation of focal adhesions and the actin cytoskeleton, integrin β3 promotes the dynamic changes necessary for cell migration, particularly during EMT. Additionally, integrin β3 modulates the expression of migration markers like N-cadherin, fibronectin, and vimentin, which contribute to enhanced cell motility [29]. These mechanisms demonstrate the pivotal role of integrin β3 in modulating TGF-β signalling and migration, making it an important target in diseases where TGF-β-driven migration and metastasis are critical, such as in cancer and fibrosis.

In this study, *ITGB3* was identified as a critical factor contributing to Trastuzumab resistance in HER2-positive breast cancer. The heterogeneous expression of *ITGB3* among resistant cell lines suggests that different subpopulations contribute variably to the resistant phenotype, reflecting the complex and multifaceted nature of resistance mechanisms. High *ITGB3* expression was associated with the upregulation of several TGF-β-responsive genes and increased cell migration, both of which are important for cancer cell survival and dissemination.

These findings align with previous research demonstrating that integrin β3 plays a key role in various cancers by promoting cell survival, migration, and invasion through multiple signalling pathways, including TGF-β. In this study, a strong correlation was observed between elevated *ITGB3* expression and increased TGF-β signalling, indicating that *ITGB3* may enhance trastuzumab resistance by modulating this pathway.

The TGF-β signalling pathway is known for its dual role in cancer: it acts as a tumour suppressor in the early stages of tumorigenesis but promotes tumour progression, metastasis, and drug resistance in later stages. The results showed that TGF-β-responsive genes, such as *WWP1*, *CARM1*, *RASGRP1*, *THBS1*, *KCTD5*, *SGCA*, *MCAM*, *FXR2*, *MTMR3*, *SOCS3*, *SLC2A4*, *MMP2*, *MMP9*, and *HSP47*, were significantly upregulated in Trastuzumab-resistant cells with high *ITGB3* expression levels, suggesting that *ITGB3* may drive the activation of TGF-β signalling and contribute to resistance.

The pronounced upregulation of these genes in clones with high *ITGB3* expression indicates that *ITGB3* may activate TGF-β signalling pathways that promote cell migration and invasion. This finding is consistent with the role of TGF-β signalling in facilitating epithelial–mesenchymal transition (EMT), a process that enhances the invasive and migratory properties of cancer cells and is often linked to therapy resistance.

The potential therapeutic benefit of combining Trastuzumab with cilengitide, an integrin inhibitor, was also explored in this study. The combination therapy effectively reduced the expression of TGF-β-responsive genes, particularly in cells with high *ITGB3* expression. This combined treatment appeared to mitigate the enhanced TGF-β signalling and migratory potential associated with high *ITGB3* levels, suggesting it could be a promising strategy to overcome resistance.

Cilengitide, which targets integrins including ITGβ3, has shown promise in preclinical studies for reducing cancer cell migration and invasion. The results of this study suggest that cilengitide can modulate integrin-mediated signalling pathways and enhance the efficacy of trastuzumab in resistant HER2-positive breast cancer cells. The observed reduction in both TGF-β-responsive and migration-related gene expression suggests that this combination therapy could inhibit the mechanisms promoting resistance and metastasis.

The correlation between *ITGB3* expression and the expression of migration-related genes (*Col4a1*, *fibronectin*, *ICAM1*, *Timp2*, and *vimentin*) further supports the role of *ITGB3* in enhancing the metastatic potential of Trastuzumab-resistant cells. The findings suggest that high ITGB3 expression is linked to increased levels of these migration markers, indicating that *ITGB3* may facilitate the invasive behaviour of resistant cells.

Interestingly, cilengitide monotherapy was more effective at reducing migration marker expression than Trastuzumab alone, suggesting that targeting *ITGB3* may directly impact the migratory behaviour of resistant cells. The combination of cilengitide with trastuzumab produced a synergistic effect, leading to the most significant reduction in migration markers, indicating that dual targeting of HER2 and ITGB3 could be an effective strategy to limit metastasis in Trastuzumab-resistant HER2-positive breast cancer.

The scratch wound healing assay revealed significant insights into how variations in ITGB3 expression influence the migratory capacity of SKBR3-R cells and their response to Trastuzumab, cilengitide, and their combination. Across all ITGB3 expression levels, control groups displayed similar wound closure percentages of approximately 85%, indicating robust baseline migratory activity. Trastuzumab monotherapy had a minimal impact on migration, with wound closure percentages remaining comparable to the DMSO control, highlighting its limited efficacy in inhibiting migration independently. In contrast, cilengitide monotherapy significantly reduced wound closure in low ITGB3-expressing cells to approximately 30% and in moderate and high ITGB3-expressing cells to 70%, suggesting that cilengitide effectively impairs migration, regardless of the ITGB3 expression level. The combination therapy demonstrated the most profound effects, reducing wound closure to as low as 5% in low ITGB3-expressing cells, 4% in moderate ITGB3-expressing cells, and 3% in high ITGB3-expressing cells. These findings indicate a synergistic effect of Trastuzumab and cilengitide in suppressing cell migration, with the combination therapy achieving significantly greater inhibition compared to either treatment alone. This observation underscores the potential of targeting integrins alongside HER2 to overcome migration-driven resistance mechanisms in trastuzumab-resistant HER2-positive breast cancer cells.

These findings highlight the potential of integrin β3 as a therapeutic target to overcome Trastuzumab resistance in HER2-positive breast cancer. By targeting ITGβ3, either alone or in combination with Trastuzumab, it may be possible to resensitize resistant cells and inhibit the pathways that promote resistance and metastasis. The combination of Trastuzumab and cilengitide presents a promising therapeutic approach that warrants further investigation in clinical trials.

This study provides valuable insights into the role of *ITGB3* in Trastuzumab resistance in HER2-positive breast cancer, but several limitations should be considered. The reliance on in vitro cell line models, such as HCC1954 and SKBR3, may not fully represent the heterogeneity of HER2-positive tumours in patients, and the lack of in vivo validation limits the translational potential of the findings. The focus on a single pathway (*ITGB3* and TGF-β signalling) may overlook other resistance mechanisms, such as PI3K/AKT or MAPK pathway alterations, which are commonly involved in Trastuzumab resistance. Additionally, this study does not examine patient-derived models, such as patient-derived xenografts (PDXs), which could offer more clinically relevant insights. The use of cilengitide, while promising, has shown mixed results in clinical trials, and this study does not explore the potential reasons for this discrepancy or strategies to enhance its effectiveness. Moreover, this study primarily focuses on *ITGB3* without addressing other potential resistance mechanisms, such as HER2 mutations or immune microenvironment changes. Finally, the method used to induce resistance, chronic exposure to increasing doses of Trastuzumab, may not fully capture the dynamic nature of resistance development in patients. Future research incorporating a broader range of models, mechanisms, and clinical data would be necessary to further validate these findings and improve therapeutic strategies.

Future research should aim to unravel the precise molecular mechanisms through which ITGB3 modulates TGF-β signalling and contributes to trastuzumab resistance. Investigating the roles of other integrins and their interactions with key signalling pathways may offer additional insights into overcoming drug resistance. Considering the heterogeneity of ITGB3 expression among resistant clones, personalized therapeutic strategies targeting specific subpopulations might be necessary. Furthermore, as gene expression changes at the mRNA level do not always correspond to protein expression or functional protein activity, validating key findings—such as ITGB3, TGF-β-responsive proteins, and migration-related proteins—through protein quantification is essential to confirm the biological relevance of the genetic data.

## 5. Conclusions

This study underscores the critical role of *ITGB3* in driving resistance to Trastuzumab and its connection to the activation of TGF-β signalling and migration-related pathways. The heterogeneous expression of *ITGB3* among resistant cell clones suggests that targeting this integrin could be an effective strategy to overcome resistance and improve outcomes in HER2-positive breast cancer. The combination of Trastuzumab with cilengitide offers a promising therapeutic approach, with the potential to achieve more effective treatments and better clinical outcomes for patients with Trastuzumab-resistant disease. Further research is required to validate these findings and explore the clinical potential of this combination therapy.

The findings from this study offer several potential clinical applications and future directions for research in overcoming Trastuzumab resistance in HER2-positive breast cancer. First, the identification of *ITGB3* as a biomarker for resistance could enable clinicians to predict and monitor Trastuzumab resistance, allowing for personalized treatment strategies. Combining Trastuzumab with cilengitide, an integrin β3 inhibitor, shows promise in resensitizing resistant tumours, and clinical trials exploring this combination could offer a new therapeutic approach. Additionally, targeting the TGF-β signalling pathway, which plays a role in resistance, could enhance treatment outcomes, particularly when combined with Trastuzumab and cilengitide. The heterogeneity of tumours underscores the need for personalized medicine, with profiling of *ITGB3* expression and TGF-β activity guiding treatment decisions. Targeting cancer stem cell properties and EMT, which are linked to resistance, could also improve patient outcomes, and investigating *ITGB3′*s role in these processes may uncover new therapeutic targets. Lastly, enhancing the efficacy of integrin inhibitors in clinical settings through combination therapies could provide a more effective strategy for overcoming resistance. Ultimately, further preclinical and clinical studies are essential to validate these approaches and optimize treatment regimens for Trastuzumab-resistant HER2-positive breast cancer patients.

## Data Availability

The datasets and materials used and/or analysed during the current study are available from the corresponding author upon reasonable request.

## Supplementary Materials

The following supporting information can be downloaded at: https://www.mdpi.com/article/10.3390/biology14010009/s1, Figure S1: ITGβ3 expression was increased in the resistant cell pool of HCC1954 and SKBR3-R cells. The figures represent the average of three independent experiments; Figure S2: *ITGB3* shows heterogeneity in resistant cell populations Heatmap shows heterogeneity of *ITGB3* expression as p values for 100 colonies from single cells for SKBR3-R and HCC1954-R cells. Two-way ANOVA with a Tukey’s post hoc test was used. Parental (P) and resistant (R) cell lines are indicated. *n* = 3; Figure S3: The TGF-β responsive genes exhibited elevated expression levels in SKBR3-R and HCC1954-R clones with high ITGB3 expression. Heatmap of TGF-B responsive gene expression levels as P values in SKBR3-R and HCC1954-R in the presence of low, moderate and high ITGB3 expression. Two-way ANOVA with a Tukey’s post hoc test was used. Parental (P) and resistant (R) cell lines are indicated. *n* = 3; Figure S4: Cilengitide monotherapy decrease TGF-B responsive expressions only low and moderate ITGB3 expressing cells while Trastuzumab+cilengitide decreased low, moderate and high ITGB3 expressing groups in SKBR3-R and HCC1954-R cells. (A) Heatmap of TGF-B responsive gene expression levels as p values in the presence of low, moderate and high ITGB3 expression in the treatment of cilengitide and trastuzumab monotherapy and combination in (A) SKBR3-R and (B) HCC1954-R. Two-way ANOVA with a Tukey’s post hoc test was used. Parental (P) and resistant (R) cell lines are indicated. *p* ≤ 0.05, *n* = 3; Figure S5: Migration markers expressions correlated with ITGB3 levels and trastuzumab+cilengitide combination significantly decrease migration markers. (A) Migration marker expression p values in low, moderate and high ITGB3 expressing cells of SKBR3-R and HCC1954-R. B) Migration marker expression *p* values in low, moderate and high ITGB3 expressing cells of SKBR3-R and HCC1954-R in the presence of cilengitide and trastuzumab monotherapy and combination. Two-way ANOVA with a Tukey’s post hoc test was used. Parental (P) and resistant (R) cell lines are indicated. *p* ≤ 0.05, *n* = 3; Table S1: Real time PCR primers used in experiments.
