# Supplementary material for: Investigation of ITGB3 Heterogeneity to Overcome Trastuzumab Resistance in HER2-Positive Breast Cancer
Source: Biology (Basel). 2024 Dec 25;14(1):9. doi: 10.3390/biology14010009 (PMC11762810; doi:10.3390/biology14010009)
Supplement: Supplementary file 1 [file biology-14-00009-s001.zip › biology-3302043-supplementary.pdf]

Supplementary Data

Supplementary Table S1: Real time PCR primers used in experiments

Gene name	Sequence	Product Length
WWP1	F: GCAGCTCATCTCCAACCATAG	201bp
	R: GAGACGGAGATGAAGGTGTG	
CARM1	F: CAGTTTTATGGCTACCTGTCCC	150bp
	R: AAACGACAGGATCCCAGAGC	
RASGM1	F: AAATCAATGCCCGTGACTGG	166bp
	R: TCAGAGAACGATATCCTCCGG	
THBS1	F: GCTCTACCAGTGTCCTCCTC	213bp
	R: TCTCTTCAGTCACTTTGCGG	
KCDT1	F: AGGAGTGTTGGAGGAAGCAG	193bp
	R: CAACTGCTCGAACTTCCAGC	
SGCA	F: CAGGTCATTGAGGTCACAGC	183bp
	R: CCCCAAGGCTGAGAGGAAG	
MCAM	F: ACTGGTTTTCTGTCCACAAGG	203bp
	R: GATGCGGTACTCCTGGGAC	
FXR2	F: GGGGATGAAGTGGAGGTTTATTC	177bp
	R: AAGGGGATTGGGATTAACTGG	
MTMR3	F: TTCCCAGGAAGCAGCTGATC	249bp
	R: ACTGACACCTGATAACTTTGCAG	
SOCS3	F: GAGAGCGGCTTCTACTGGAG	162bp
	R: CTGGATGCGCAGGTTCTTG	
SLC2A4	F: AGAGCCACCCCAGGAAAAG	225bp
	R: CGGAGAGGACTGGTCACTG	
MMP2	F: CAGGATCATTGGCTACACACC	151bp
	R: CCAGCGGCCAAAGTTGATC	
MMP9	F: TGGATCCAAAACTACTCGGAAG	250bp
	R: CATCGTCGAAATGGGCGTC	
PAI1	F: AGACCGATTATTGACCGACC	210bp
	R: CCAGTTGTGAGATCCGCTAC	
HSP47	F: CGCCATGTTCTTCAAGCCAC	244bp
	R: CTTTTCAAGGCGCTCGAGAG	

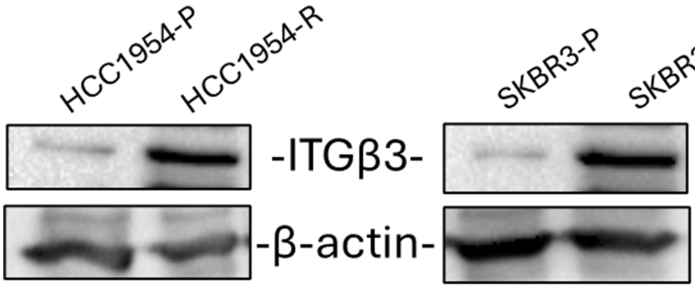

Figure S1: ITGβ3 expression was increased in the resistant cell pool of HCC1954 and SKBR3-R cells. The figures represent the average of three independent experiments.

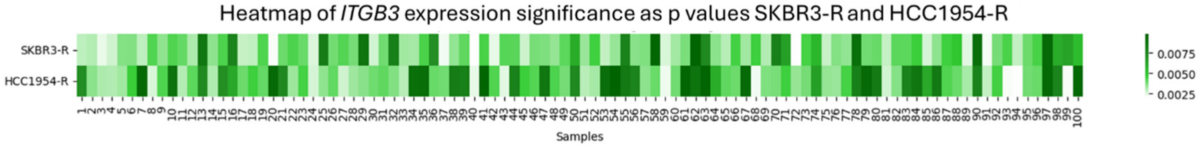

Figure S2: *ITGB3* shows heterogeneity in resistant cell populations Heatmap shows heterogeneity of *ITGB3* expression as p values for 100 colonies from single cells for SKBR3-R and HCC1954-R cells. Two-way ANOVA with a Tukey's post hoc test was used. Parental (P) and resistant (R) cell lines are indicated. n = 3

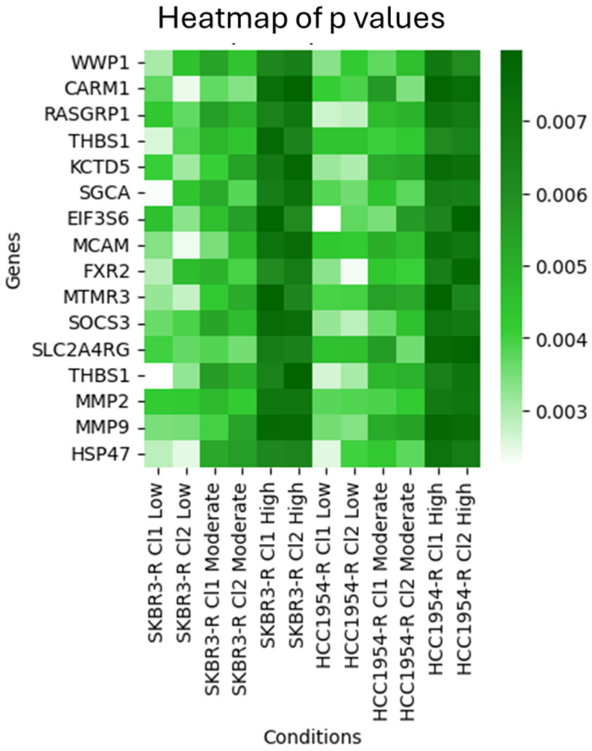

Figure S3: The TGF-β responsive genes exhibited elevated expression levels in SKBR3-R and HCC1954-R clones with high ITGB3 expression. Heatmap of TGF-B responsive gene expression levels as P values in

SKBR3-R and HCC1954-R in the presence of low, moderate and high ITGB3 expression. Two-way ANOVA with a Tukey's post hoc test was used. Parental (P) and resistant (R) cell lines are indicated. n = 3

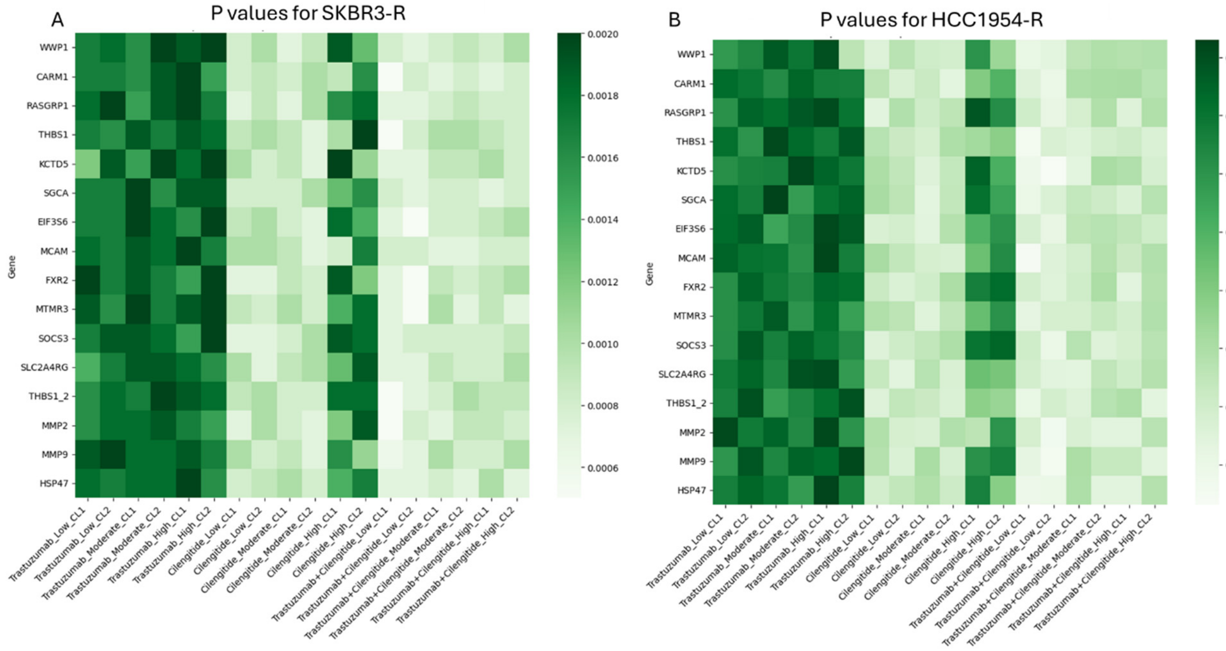

Figure S4: Cilengitide monotherapy decrease TGF-B responsive expressions only low and moderate ITGB3 expressing cells while Trastuzumab+cilengitide decreased low, moderate and high ITGB3 expressing groups in SKBR3-R and HCC1954-R cells. A) Heatmap of TGF-B responsive gene expression levels as p values in the presence of low, moderate and high ITGB3 expression in the treatment of cilengitide and trastuzumab monotherapy and combination in A) SKBR3-R and B) HCC1954-R. Two-way ANOVA with a Tukey's post hoc test was used. Parental (P) and resistant (R) cell lines are indicated. *p* ≤ 0.05, n = 3

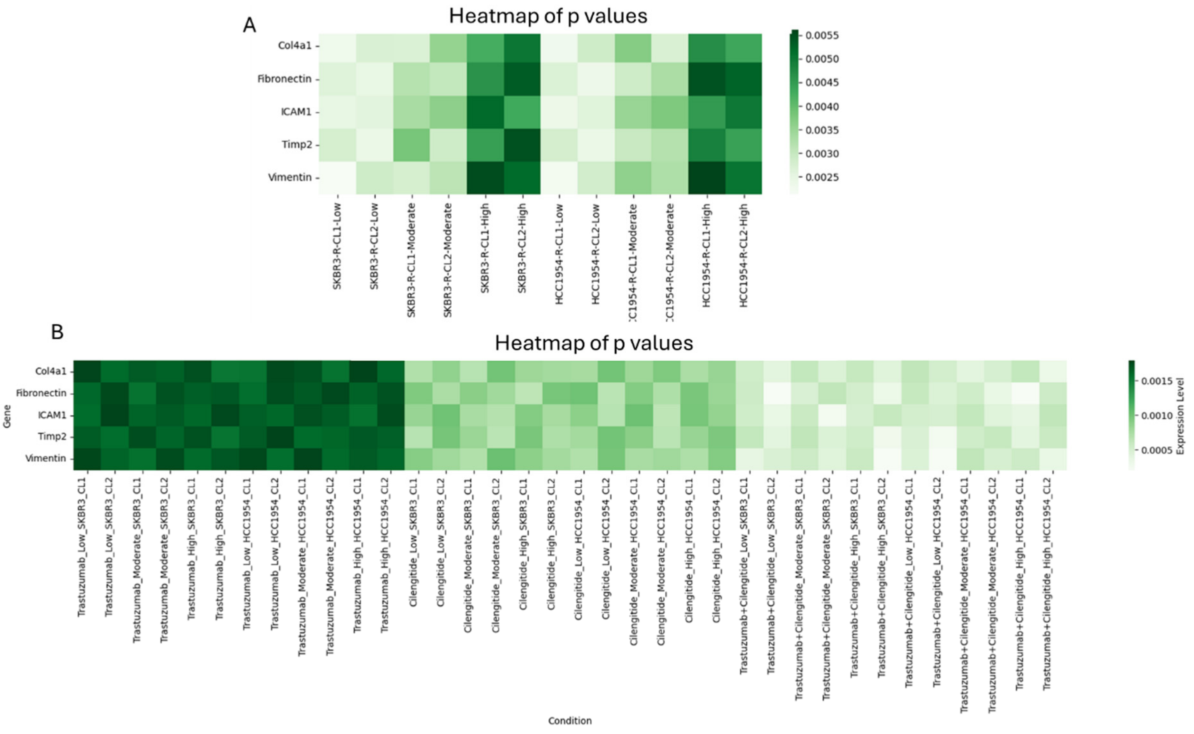

Figure S5: Migration markers expressions correlated with ITGB3 levels and trastuzumab+cilengitide combination significantly decrease migration markers. A) Migration marker expression p values in low, moderate and high ITGB3 expressing cells of SKBR3-R and HCC1954-R. B) Migration marker expression p values in low, moderate and high ITGB3 expressing cells of SKBR3-R and HCC1954-R in the presence of cilengitide and trastuzumab monotherapy and combination. Two-way ANOVA with a Tukey's post hoc test was used. Parental (P) and resistant (R) cell lines are indicated. $p \leq 0.05$, n = 3
